# Clinical and immunological features of rheumatoid arthritis patients with positive anti-centromere antibody: a case-control study

**DOI:** 10.3389/fmed.2026.1882422

**Published:** 2026-07-30

**Authors:** Xiuqing Yan, Meili Wang, Fengmei Ge, Linyi Peng, Xuebin Wang, Li Wang

**Affiliations:** 1 Department of Rheumatology and Clinical Immunology, Peking Union Medical College Hospital, Chinese Academy of Medical Sciences & Peking Union Medical College, Beijing, China; 2 Department of Rheumatology and Clinical Immunology, Shandong Medical and Pharmaceutical University Hospital, Binzhou, Shandong, China; 3 Department of Rheumatology and Clinical Immunology, Ji’an Central People’s Hospital, Ji’an, Jiangxi, China

**Keywords:** anti-centromere antibody, clinical characteristics, CREST syndrome, interstitial lung disease, rheumatoid arthritis

## Abstract

**Objective:**

To characterize the clinical and immunological features of rheumatoid arthritis (RA) patients with positive anti-centromere antibody (ACA).

**Methods:**

In this retrospective case-control study, the clinical manifestations, demographic data, and immunological test results of these patients were collected and analyzed.

**Results:**

ACA-positive RA patients exhibited a significantly higher prevalence of Raynaud’s phenomenon (15.0% vs. 2.5%, *χ*^2^ = 4.838, *P* = 0.028), xerostomia (22.5% vs. 8.8%, *χ*^2^ = 4.363, *P* = 0.037), xerophthalmia (27.5% vs. 10.0%, *χ*^2^ = 6.128, *P* = 0.013), and interstitial pneumonia (27.5% vs. 5.0%, *χ*^2^ = 12.343, *P* < 0.001), as well as higher rates of elevated ALP (18.9% vs. 1.3%, *χ*^2^ = 9.780, *P* = 0.002) and GGT (33.33% vs. 15.0%, *χ*^2^ = 5.086, *P* = 0.024).

**Conclusion:**

ACA-positive RA may present a unique phenotype characterized by an increased risk of interstitial lung disease, sicca symptoms, and cholestatic liver injury; thus, enhanced pulmonary/hepatic screening and tailored monitoring strategies are critical.

## Introduction

Rheumatoid arthritis (RA) is an autoimmune disease primarily characterized by erosive polyarthritis, representing one of the most common causes of disability in the general population (1). Anti-centromere antibody (ACA) is a subtype of antinuclear antibody that specifically targets centromere antigens (2) and is generally recognized as a serological marker for CREST syndrome (Calcinosis, Raynaud’s phenomenon, Esophageal dysmotility, Sclerodactyly, Telangiectasia) (3). Beyond CREST syndrome, recent studies have linked ACA to other autoimmune conditions, such as systemic lupus erythematosus (4), Sjögren’s syndrome (5), primary biliary cholangitis (6), and even RA. Limited data have demonstrated that ACA-positive RA patients may have older age at onset, female predominance, longer disease duration, and an increased prevalence of Raynaud’s phenomenon (7, 8). But whether it exhibits the distinct clinical features and immunological phenotypes remains unclear. We summarized the clinical characteristics of ACA-positive RA patients and compared them with other RA patients to provide preliminary evidence for more precise diagnosis and management.

## Materials and methods

### Study subjects

We adopted a retrospective case–control design. Forty ACA-positive RA patients and eighty matched control patients in terms of gender, age, and disease duration who were treated in the Department of Rheumatology and Clinical Immunology of Peking Union Medical College Hospital from January 2020 to January 2025 were included. All patients met the 2010 ACR/EULAR RA classification criteria (9) or the 1987 classification criteria of the American College of Rheumatology (ACR) (10), with their diagnoses confirmed by two senior rheumatologists through comprehensive clinical assessments.

### Data collection and laboratory measurements

Demographic and baseline clinical variables, including age, gender, disease duration, and the 28-joint Disease Activity Score using erythrocyte sedimentation rate (DAS28-ESR) (11), were extracted from electronic medical records. Clinical manifestations (xerostomia, xerophthalmia, Raynaud’s phenomenon) and complications were collected via manual review. Although some patients were positive for anti-SSA antibody, none met the diagnostic criteria for primary Sjögren’s syndrome (pSS). Interstitial lung disease (ILD) was diagnosed by high-resolution computed tomography (HRCT) based on typical clinical and radiologic features after excluding other etiologies (12). Only RA-associated ILD was analyzed. Serum autoantibodies were measured using standardized protocols at Peking Union Medical College Hospital. ACA was detected by iFlash Centromere IgG chemiluminescence immunoassay following the manufacturer’s standard protocol. Per kit instructions, the cut-off value for ACA positivity was set at 20.00 AU/mL; specimens with concentrations < 20.00 AU/mL were classified as ACA-negative, while values ≥ 20.00 AU/mL were defined as ACA-positive. The assay displayed a linear detection range of 2.00–2000.00 AU/mL with acceptable repeatability and batch-to-batch precision. RF, anti-CCP, and ANA (detected via indirect immunofluorescence; positivity defined as a titer ≥ 1:100) were tested using a digital liquid chip assay. Serum levels of anti-ENA, ANCA, ACL, anti-Ro52, anti-SSA60, anti-SSB, AMA-M2, and anti-Scl70 were quantitatively determined with commercial chemiluminescent immunoassay (CLIA) kits. Routine laboratory parameters, including ESR, CRP, immunoglobulins, hepatic and renal function, and complete blood count, were measured from fasting venous blood samples in accordance with standard operating procedures.

To visually characterize the overlapping distribution of multiple autoantibodies among ACA-positive RA patients and identify distinct immunological subgroups, an autoantibody co-positivity heatmap was constructed based on binary positive/negative results of all tested autoantibodies; all references to Pearson correlation were removed from the figure description as recommended, given that descriptive heatmap only quantifies pairwise autoantibody co-occurrence frequencies rather than continuous correlation analysis. This graphical tool has two auxiliary clinical applications: first, it stratifies ACA-positive RA patients into isolated ACA-positive and multi-autoantibody co-positive subgroups to compensate for the limitation that single ACA quantification fails to reflect comprehensive autoimmune profiles; second, it enables preliminary exploration of associations between overlapping autoantibodies and extra-articular complications, including interstitial lung disease and sicca symptoms. Importantly, this heatmap merely provides supplementary descriptive data and cannot be regarded as core statistical evidence supporting our primary findings related to ACA positivity and RA-ILD.

### Statistical analysis

Data analysis was performed using SPSS 26.0. Enumeration data were presented as frequency and percentage (*n*, %). Measurement data conforming to a normal distribution were expressed as the mean ± standard deviation (Mean ± SD), whereas non-normally distributed variables were expressed as the median and interquartile range (Median, IQR). For group comparisons, the chi-square test (*χ*^2^ test) or Fisher’s exact test was used for enumeration data. The independent samples *t*-test was used to compare normally distributed measurement data between two groups. The Mann–Whitney *U* test was used for ranked data and non-normally distributed measurement data. Univariate variables with statistical significance were included in binary logistic regression analysis. The significance level α was set at 0.05, and a *P* value < 0.05 was considered statistically significant.

Given the retrospective design relying on historical electronic medical records, missing values were observed in several clinical indicators. To guarantee the robustness of statistical outcomes, each analysis was performed based on the actual valid sample with complete data for the corresponding variables, rather than the overall total cohort. No data imputation or arbitrary exclusion of participants was conducted in this study.

## Results

### Comparison of baseline characteristics and clinical phenotypes between ACA-positive RA and the control group

**FIGURE 1:**
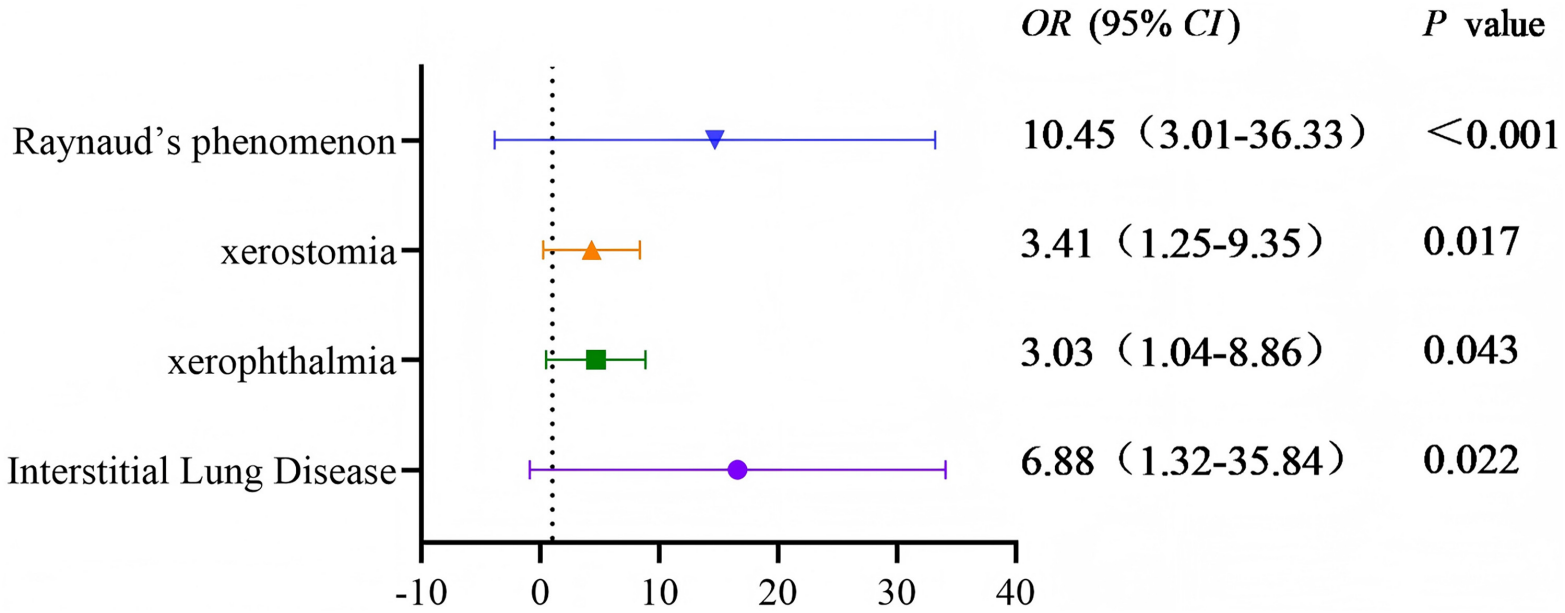
Odds ratios (ORs) for the association between clinical symptoms and ACA positivity: Results of binary logistic regression. This forest plot presents the odds ratios (ORs) and 95% confidence intervals (CIs) for the associations between clinical symptoms and anti-centromere antibody (ACA) positivity. An OR > 1 indicated a positive association between the symptom and ACA positivity. A *P*-value < 0.05 was considered statistically significant.

A total of 120 RA patients were enrolled, including ACA-positive RA patients (*n* = 40) and ACA-negative RA patients (*n* = 80, control group). There were no statistically significant differences in age (59.9 ± 10.93 years vs. 58.59 ± 11.01 years, *t* = 0.617, *P* = 0.538), gender distribution (95.0% female in both groups, *χ*^2^ = 0.000, *P* = 1.000), or disease duration [6.0 (1.0–10.0) years vs. 4.0 (1.13–8.75) years, *Z* = 0.592, *P* = 0.554] between the two groups, indicating good comparability (Table 1). Compared with the control group, the ACA-positive RA group exhibited a significantly higher prevalence of Raynaud’s phenomenon (15.0% vs. 2.5%, *χ*^2^ = 4.838, *P* = 0.028), xerostomia (22.5% vs. 8.8%, *χ*^2^ = 4.363, *P* = 0.037), and xerophthalmia (27.5% vs. 10.0%, *χ*^2^ = 6.128, *P* = 0.013). There was a significant unadjusted association between ACA positivity and ILD in our cohort (Table 1). Among the 11 ILD cases in the ACA-positive RA group, 7 were nonspecific interstitial pneumonia (NSIP), 2 were usual interstitial pneumonia (UIP), and 1 had mixed NSIP/UIP; the control group had 4 ILD cases, all of which were NSIP. Additionally, no significant differences were observed in sclerodactyly between the two groups. Calcinosis, telangiectasia, and esophageal dysmotility were absent in both groups (prevalence = 0%, statistical analysis not applicable [N/A]) (Table 1). Binary logistic regression analysis confirmed that ACA positivity was independently associated with an increased risk of these clinical phenotypes (after adjusting for age, gender, and disease duration), with respective odds ratios (ORs) and 95% confidence intervals (CIs) as follows: Raynaud’s phenomenon (OR = 10.45, 95% CI: 3.01–36.33, *P* < 0.001), xerostomia (OR = 3.41, 95% CI: 1.25–9.35, *P* = 0.017), xerophthalmia (OR = 3.03,95% CI: 1.04–8.86, *P* = 0.043), and ILD (OR = 6.88, 95% CI: 1.32–35.84, *P* = 0.022) (Figure 1).

**TABLE 1:** Comparison of baseline characteristics and clinical phenotypes between ACA-positive RA and the control group.

Baseline characteristics/clinical phenotypes	ACA-positive RA (*n* = 40)	control group (*n* = 80)	Statistic	*P-*value
Age (years)	59.9 ± 10.93	58.59 ± 11.01	*t* = 0.617	0.538
Gender, *n* (%)			*χ*^2^ = 0.000	1.000
—Man	2 (5.0%)	4 (5.0%)		
—Women	38 (95.0%)	76 (95.0%)		
disease duration (years)	6.0 (1.0, 10.0)	4 (1.13, 8.75)	*Z* = 0.592	0.554
Raynaud’s phenomenon, *n* (%)	6 (15.0%)	2 (2.5%)	*χ*^2^ = 4.838	0.028
sclerodactyly, *n* (%)	1 (2.5%)	1 (1.3%)	N/A	N/A
calcinosis, *n* (%)	0	0	N/A	N/A
telangiectasia, *n* (%)	0	0	N/A	N/A
Esophageal Dysmotility, *n* (%)	0	0	N/A	N/A
xerostomia, *n* (%)	9 (22.5%)	7 (8.8%)	*χ*^2^ = 4.363	0.037
xerophthalmia, *n* (%)	11 (27.5%)	8 (10.0%)	*χ*^2^ = 6.128	0.013
Interstitial lung disease, *n* (%)	11 (27.5%)	4 (5.0%)	*χ*^2^ = 12.343	< 0.001

Data are presented as mean ± SD, median (IQR), or *n* (%). Intergroup comparisons used the *t*-test/Wilcoxon test/*χ*^2^ test (as appropriate). *P* < 0.05 was considered statistically significant. ACA-positive RA, anti-centromere antibody-positive RA; control group, anti-centromere antibody-negative RA; N/A, Not applicable (no data available for statistics analysis).

### Comparison of autoantibodies and immunological indicators between the two groups

**FIGURE 2:**
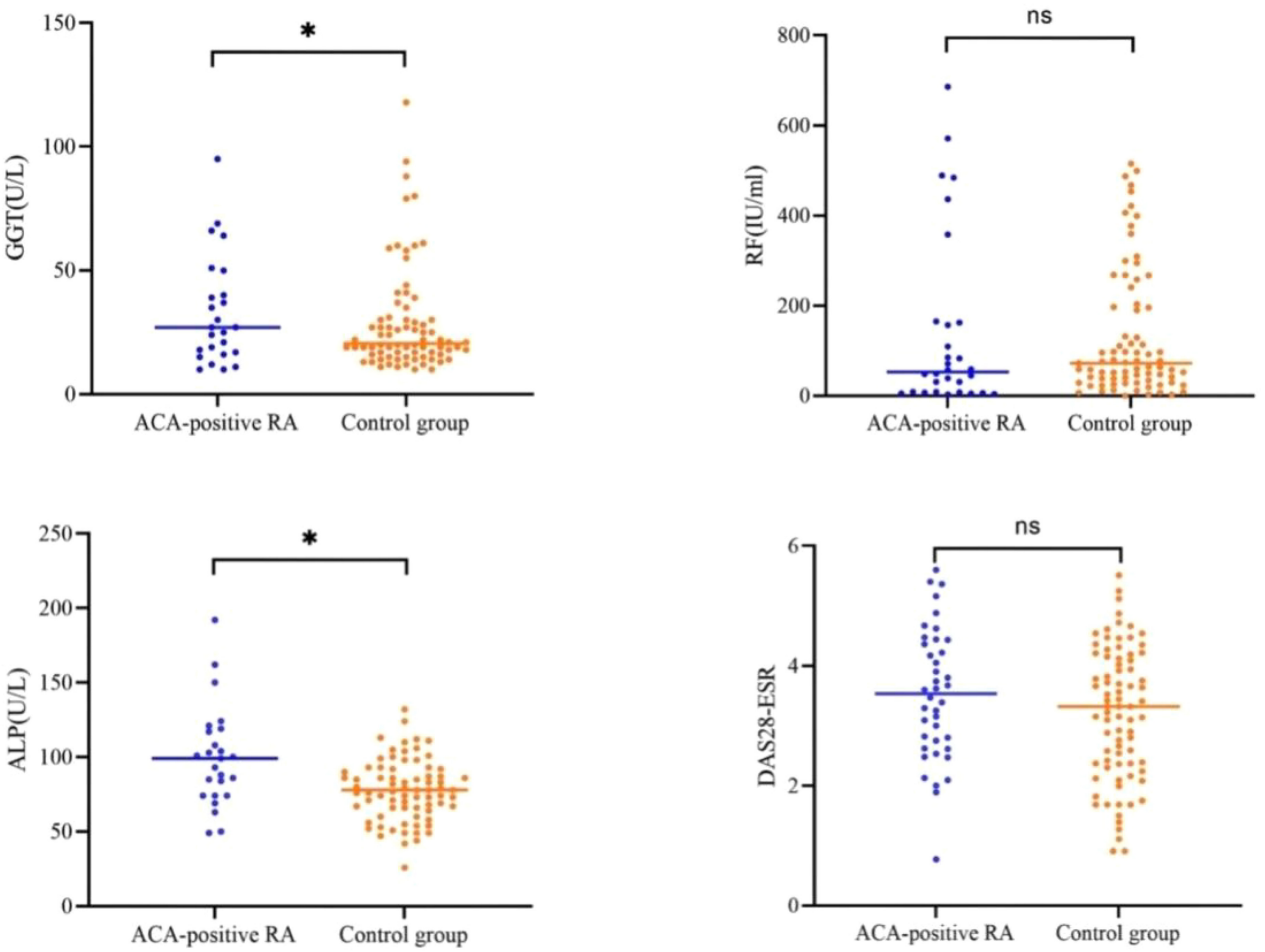
Scatter Plot of GGT, ALP, RF, and DAS28 Levels Between ACA++RA and ACA-RA Patients. This figure shows the levels of four indicators in ACA-positive RA (blue dots) and ACA-negative RA (orange dots) patients: GGT (U/L), ALP (U/L), RF (U/mL), and DAS28. Horizontal lines represent the median values. Statistical significance: *indicates *P* < 0.05; ns indicates *P* ≥ 0.05 (not significant).

**FIGURE 3:**
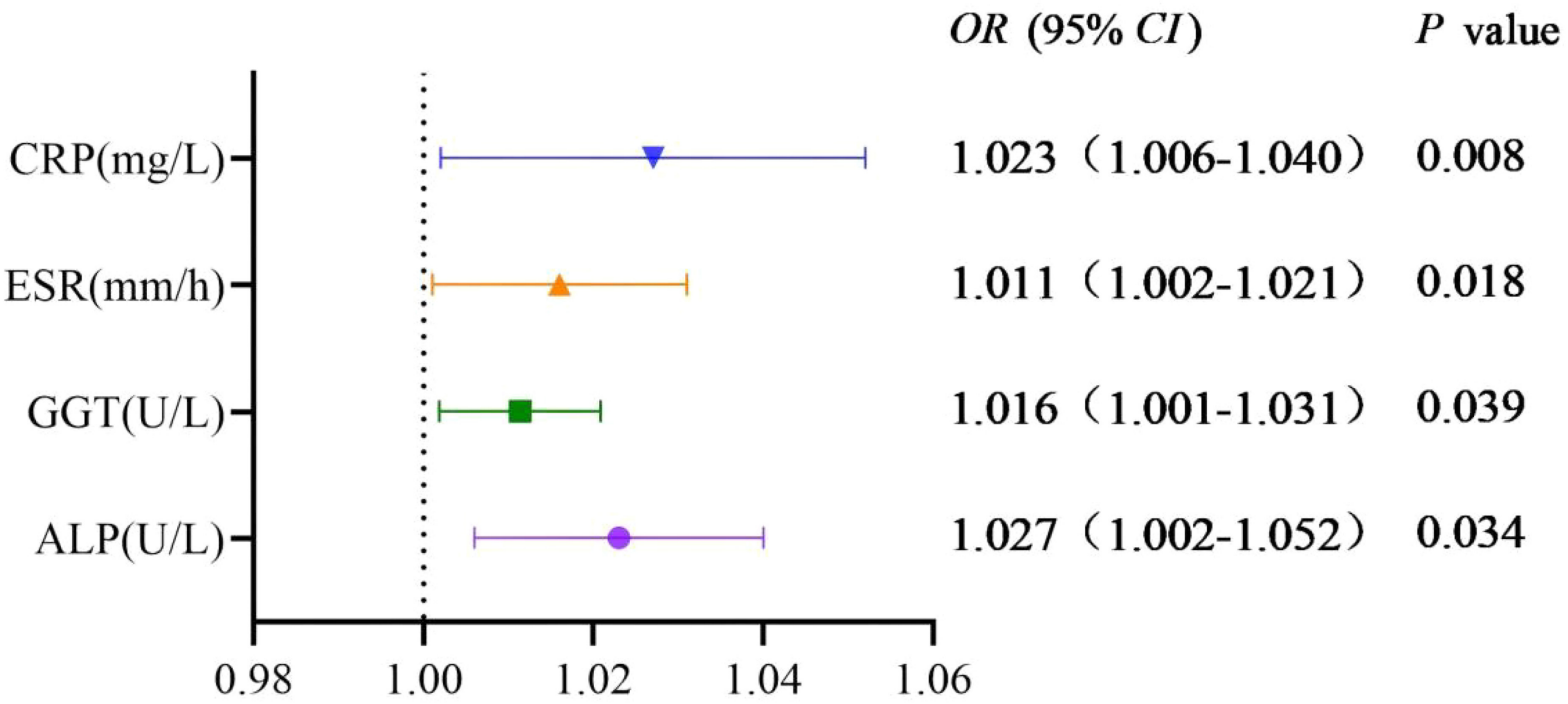
Factors associated with ACA positivity: results of binary logistic regression. This forest plot shows the odds ratios (ORs) and 95% confidence intervals (CIs) for the associations between laboratory indices (CRP, ESR, GGT, ALP) and ACA positivity. An OR > 1 indicates that higher values of the index were associated with an increased likelihood of ACA positivity. The dashed vertical line represents the null effect (OR = 1.00). Statistical significance was defined as *P* < 0.05.

Compared with the control group, the ACA-positive RA group had a significantly higher prevalence of high ANA titer ( > 320) (72.5% vs. 11.3%, *χ*^2^ = 46.232, *P* < 0.001). Regarding immunological and biochemical indicators, the ACA-positive RA group exhibited significantly elevated ESR [28.0 (14.5–45.5) mm/h vs. 18.0 (9.0–30.5) mm/h, *Z* = 2.349, *P* = 0.019] and CRP [3.93 (1.95–15.82) mg/L vs. 1.82 (0.71–6.31) mg/L, *Z* = 2.658, *P* = 0.008], as well as higher rates of elevated gamma-glutamyl transferase (GGT) (33.3% vs. 15.0%, *χ*^2^ = 5.086, *P* = 0.024) and alkaline phosphatase (ALP) (18.9% vs. 1.3%, *χ*^2^ = 9.780, *P* = 0.002). Additionally, the prevalence of elevated IgG (22.2% vs. 6.3%, *χ*^2^ = 4.861, *P* = 0.027) and IgM (18.4% vs. 2.5%, *χ*^2^ = 7.147, *P* = 0.008) was significantly higher in the ACA-positive RA group. In contrast, no significant differences were found in leukocyte count, hemoglobin level, platelet count, alanine aminotransferase (ALT), aspartate aminotransferase (AST), RF titer, anti-CCP antibody positive rate, or DAS28-ESR between the two groups (all *P* > 0.05) (Figure 2 and Table 2). Binary logistic regression analysis revealed that higher levels of CRP, ESR, GGT, and ALP were associated with ACA positivity (all *P* < 0.05) (Figure 3).

**TABLE 2:** Comparison of autoantibodies and laboratory parameters between ACA-positive RA and the control group.

Items	Positive/available in ACA-positive RA n (*n* %)	Positive/available in Control group n (*n* %)	Statistic *χ*^2^/*t/Z*	*P-*value
High ANA titer ( > 320), *n* (%)	25/40 (62.5%)	9/80 (11.3%)	46.232	< 0.001
Anti-CCP, *n* (%)	25/37 (67.6%)	64/80 (81.0%)	2.551	0.110
Elevated RF, *n* (%)	26/36 (72.2%)	69/80 (86.3%)	3.295	0.069
Elevated IgG, *n* (%)	8/36 (22.2%)	5/80 (6.3%)	4.861	0.027
Elevated IgM, *n* (%)	7/38 (18.4%)	2/80 (2.5%)	7.147	0.008
Elevated IgA, *n* (%)	6/35 (17.1%)	5/80 (6.3%)	2.199	0.138
Elevated ALT, *n* (%)	7/40 (17.5%)	11/80 (13.8%)	0.360	0.548
Elevated AST, *n* (%)	7/37 (18.9%)	9/80 (11.3%)	0.798	0.372
Elevated GGT, *n* (%)	12/36 (33.33%)	12/80 (15.0%)	5.086	0.024
Elevated ALP, *n (%*)	7/37 (18.9%)	1/77 (1.3%)	9.780	0.002
Leukocyte/(10^9^/L)	5.91 ± 1.95	6.48 ± 2.14	1.358	0.177
Hemoglobin/(g/L)	124.03 ± 13.90	124.04 ± 16.45	0.003	0.998
Platelet/(10^9^/L)	234.34 ± 86.27	255.59 ± 76.25	1.318	0.190
ESR, M (Q1, Q3)	28.0 (14.5, 45.5)	18.0 (9.0, 30.5)	−2.349	0.019
CRP, M (Q1, Q3)	3.93 (1.95, 15.82)	1.82 (0.71, 6.31)	−2.658	0.008
RF, M (Q1, Q3)	53.0 (9.0, 160.98)	73.60 (36.25, 222.20)	−1.082	0.279
DAS28-ESR, Mean ± SD	3.52 ± 1.09	3.20 ± 1.11	1.481	0.141

For categorical indicators, values were presented as the number of positive cases/available cases (%) with percentages calculated from participants with non-missing data. Continuous variables were expressed as the mean ± standard deviation or median (interquartile range), depending on the data distribution, and including only individuals with valid data. The χ^2^ test was applied for categorical variables, while the independent-samples *t*-test or Mann-Whitney U-test (Z-test) was used for continuous variables. *P* < 0.05 was considered statistically significant. ANA, antinuclear antibody; Anti-CCP, anti-cyclic citrullinated peptide antibody; RF, rheumatoid factor; IgG, immunoglobulin G; IgM, immunoglobulin M; IgA, immunoglobulin A; ALT, alanine aminotransferase; AST, aspartate aminotransferase; GGT, gamma-glutamyl transferase; ALP, alkaline phosphatase; ESR, erythrocyte sedimentation rate; CRP, C-reactive protein; M (Q1,Q3), Median (1st Quartile, 3rd Quartile); DAS28-ESR, Disease Activity Score in 28 Joints-erythrocyte sedimentation rate; SD, standard deviation.

### Analysis of positive co-occurrence characteristics between autoantibodies and clinical phenotypes/laboratory indicators

**FIGURE 4:**
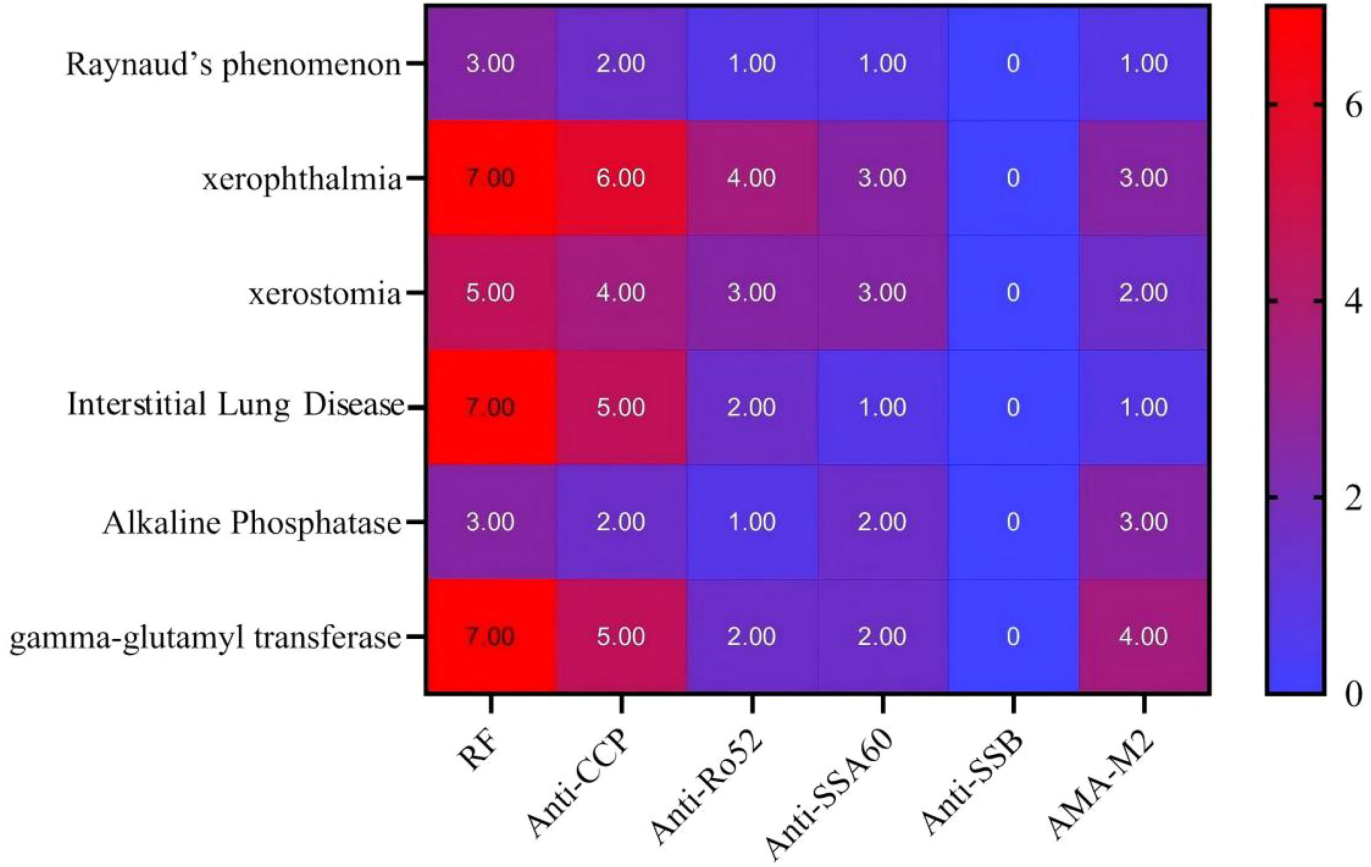
Co-positivity heatmap of autoantibodies and clinical-laboratory manifestations in ACA+RA patients. Heat map showing the number of patients with concurrent positivity for autoantibodies and clinical-laboratory parameters in patients with ACA-positive RA. Color intensity corresponds to the count of patients with co-positivity for each autoantibody-parameter pair, and the actual case numbers are presented inside individual cells.

Co-positivity patterns between autoantibodies and clinical-laboratory indices among patients with ACA-positive RA were quantified and visualized via a heatmap (Figure 4). The color gradient represented the number of patients with concurrent positivity for each autoantibody-phenotype pair, ranging from dark blue for zero co-positive patients to dark red for a maximum of seven co-positive patients. Exact numbers of matched subjects were annotated within each cell for intuitive reading. According to the heat-map-derived descriptive data, the combinations of RF together with xerophthalmia, interstitial lung disease (ILD), and elevated GGT showed the highest number of co-positive patients (*n* = 7). The pairing of anti-CCP and xerophthalmia exhibited the second-highest co-positive case number (*n* = 6). Notably, anti-SSB presented no co-occurrence with any of the evaluated clinical-laboratory parameters in this ACA-positive RA cohort. In addition, Raynaud’s phenomenon had relatively limited co-occurrence with the detected autoantibodies, with a maximum of only three co-positive patients across all antibody-phenotype pairs. Collectively, these descriptive findings revealed that RF and anti-CCP displayed prominent co-existing status with key clinical manifestations and laboratory abnormalities among ACA-positive RA patients, whereas anti-SSB failed to show such co-positivity with the examined indicators.

## Discussion

ACA is classically recognized as the hallmark autoantibody of CREST syndrome. Emerging evidence has indicated that ACA may participate in the pathogenesis of RA, although the underlying mechanisms remain not fully elucidated (13). Notably, no typical CREST-associated cutaneous manifestations were observed in our ACA-positive RA cohort. These findings support a dissociation between ACA positivity and classic CREST syndrome-related features in patients with RA, which is consistent with the observations reported by Jearn et al. (13). A potential explanation for this dissociation may lie in the divergent functional roles of ACA across different connective tissue diseases. The major target antigen of ACA is centromere protein B (CENP-B). In systemic sclerosis (SSc), ACA act as disease-specific autoantibodies closely linked to vascular damage and fibrosis. In contrast, ACA may serve as disease-associated autoantibodies in RA. Since CENP-B has limited relevance to the core pathological processes of synovial inflammation and joint destruction that characterize RA, it is unlikely to trigger the profibrotic signaling cascades that drive CREST syndrome phenotypes. As autoantibodies targeting chromosomal centromere proteins, ACA in RA may likely reflect systemic immune homeostasis disturbance rather than direct tissue injury. The development of fibrosis and vascular injury—hallmarks of CREST syndrome—may likely require additional pathogenic drivers, including endothelial cell apoptosis and transforming growth factor-β (TGF-β) pathway activation (14, 15). Collectively, these observations suggest that ACA may represent a risk marker for SSc rather than a direct driver of CREST-like phenotypes in RA. Clinically, this supports the use of ACA positivity as a phenotypic stratification tool for differential diagnosis: ACA-positive patients presenting with Raynaud’s phenomenon and skin thickening should be prioritized for evaluation for SSc or connective tissue disease overlap syndrome. Whereas those with typical RA manifestations and no CREST-related features can be appropriately diagnosed as ACA-positive RA.

This study found that the prevalence of ILD in ACA-positive RA patients was 27.5%, significantly higher than that in ACA-negative RA patients. However, while we observed a correlation between ACA positivity and ILD, this finding should be interpreted cautiously given the absence of adjustment for smoking history, RA treatment, and comorbidities that may act as strong confounders. Notably, this rate is substantially higher than the 11.8% ILD prevalence reported in prior ACA-positive RA cohorts (13). This discrepancy may relate to differences in sample size, ILD diagnostic criteria (e.g., more widespread HRCT use in our study), or patient demographic characteristics. Furthermore, our observed rate exceeds both the pooled ILD prevalence (18.7%) in the general RA population and the 22.9% prevalence in HRCT-diagnosed RA-ILD cohorts from a recent meta-analysis (16). These comparisons highlight that ACA-positive RA confers elevated ILD risk, distinct from ACA-negative RA and noteworthy even against general RA or HRCT-diagnosed subgroups. This finding aligns with the cohort results published by Kuramoto et al. (8), and collectively, these data support the existence of a statistical association between ACA positivity and ILD in RA patients. From a mechanistic perspective, chronic synovial inflammation in RA may lead to the release of autoantigens (e.g., citrullinated proteins and CENP-B), which could trigger cross-reactive immune responses targeting lung tissue via molecular mimicry. Such responses may subsequently activate lung fibroblasts and promote the progression of pulmonary fibrosis (17, 18). Furthermore, ILD in ACA-positive RA patients in this study was predominantly of the nonspecific interstitial pneumonia (NSIP) subtype (7/11 cases), which differs from most previous literature reporting usual interstitial pneumonia (UIP) as the most common pathological pattern in RA-associated ILD (19). This preliminary observation suggests that ACA may potentially influence the subtype distribution of ILD in RA. This hypothesis requires further validation in larger cohorts. Binary logistic regression analysis in this study demonstrated a positive association between ACA positivity and ILD in RA. As such, baseline HRCT and pulmonary function testing, combined with regular long-term monitoring, may be recommended for ACA-positive RA patients during clinical follow-up to enable early detection and intervention for ILD.

Our study showed that ACA-positive RA patients had markedly higher prevalences of xerostomia (22.50%) and xerophthalmia (27.50%) than ACA-negative cases, with ACA positivity identified as having an independent association with both sicca symptoms after adjusting for confounding factors. Notably, none of the ACA-positive RA patients with partial anti-SSA positivity met pSS diagnostic criteria, and anti-SSB antibodies had no co-positivity with sicca symptoms, indicating that these symptoms are unique phenotypes of this subgroup rather than comorbid pSS, further validating the distinct immunological and clinical profile of ACA-positive RA (8). This phenotypic feature aligns with findings in ACA-positive pSS patients who present with mild, persistent sicca symptoms without severe labial gland lymphocytic infiltration (5). Mahajan et al. (20) further confirmed that ACA-mediated exocrine gland injury is characterized by low-grade chronic inflammation rather than severe structural damage. Kuramoto et al. (8) reported a 21.3% incidence of sicca symptoms in ACA-positive RA patients, which frequently coexisted with Raynaud’s phenomenon and ILD, consistent with the concurrent elevation of these manifestations in our cohort. Together, these results confirm a cross-disease consistent phenotype of ACA-mediated exocrine gland injury characterized by low-grade chronic inflammation and secretory dysfunction without severe tissue infiltration (20), and these findings offer a key basis for clinically differentiating ACA-positive RA with sicca symptoms from RA complicated with secondary pSS. Immunologically, sicca symptoms in ACA-positive RA arise from multi-dimensional immune dysregulation. Abnormal humoral immune activation with elevated IgG and IgM levels underpins exocrine gland injury (13), and ACA may synergize with RF and anti-CCP—both showing the highest co-positivity with xerophthalmia—to exacerbate exocrine gland immune injury via cross-reactive immune responses based on the antigen-epitope competition hypothesis (13).

A comparison of autoantibody profiles between ACA-positive and ACA-negative RA patients in this study revealed a significantly higher rate of high ANA titers in the ACA-positive group, a finding that may be related to the inherent immunogenicity of ACA. The positive rates of RF and anti-CCP antibodies were slightly lower in ACA-positive RA patients relative to the ACA-negative control group, which is consistent with the antigen-epitope competition hypothesis proposed by Jearn et al. (13). The chronic inflammatory microenvironment in RA may lead to the exposure of multiple self-antigens, and structural similarities between these antigens may trigger cross-immune reactions via molecular mimicry, thereby potentially inducing ACA production (16, 21). The current study also observed numerically higher ESR, CRP levels, and DAS28-ESR scores in ACA-positive RA patients; however, no statistically significant intergroup difference was identified (*P* = 0.141). This mild numerical trend alone cannot serve as evidence of stronger systemic inflammation in this subgroup. Long-term high disease activity in RA is known to potentially drive epitope spreading, which may contribute to the development of more complex clinical phenotypes. This observed serological “separation phenomenon” may tentatively imply that ACA positivity in RA is associated with a distinct immune activation pathway, though further mechanistic studies are needed to confirm this hypothesis. Additionally, analysis of immune and liver function indices further identified significant differences in IgM and IgG levels between ACA-positive and ACA-negative RA patients, suggesting that ACA positivity may be associated with enhanced humoral immune activation in RA. This enhanced activation may increase the potential risk of immune complex-mediated disease activity, though this speculative association merits further investigation. In terms of liver function parameters, ALP and GGT levels were significantly elevated in ACA-positive RA patients and were found to be independently associated with ACA positivity, while no significant differences in ALT or AST levels were observed between the two groups. These findings indicate that ACA-positive RA patients may be more prone to cholestatic liver injury, a unique phenotypic feature that deserves careful attention in clinical management.

The co-occurrence analysis revealed distinct concomitant correlations between specific autoantibodies and key clinical/laboratory features in ACA-positive RA patients, further supporting the unique immunological phenotype of this subgroup. Notably, RF and anti-CCP—classic serological markers of RA—exhibited robust co-positivity with xerophthalmia, ILD, and elevated GGT, with RF reaching the highest co-occurrence count (*n* = 7) when paired with xerophthalmia, ILD, and elevated GGT. This finding aligns with the well-established role of RF and anti-CCP in reflecting systemic immune activation in RA, as their co-occurrence with sicca symptoms (xerophthalmia), pulmonary involvement, and cholestatic liver injury (reflected by elevated GGT) may indicate a more extensive autoimmune response beyond synovial inflammation. Mechanistically, the concurrent presence of these autoantibodies and organ-specific manifestations could be attributed to shared autoantigenic targets or cross-reactive immune pathways. Clinically, these co-occurrence patterns offer valuable insights for risk stratification and differential diagnosis in ACA-positive RA. The robust co-association of RF/anti-CCP with ILD and elevated GGT implies that ACA-positive RA patients positive for RF or anti-CCP have a greater likelihood of pulmonary and hepatic involvement, warranting more intensive screening for these manifestations.

### Limitations

This study has three main limitations. First, only 40 ACA-positive RA patients were enrolled in our single-center cohort; the small sample size lowered statistical power and rendered multivariable regression results less stable. Second, retrospective medical record data lacked complete information on smoking history, RA medications, disease activity, and comorbidities. Without adjustment for these important confounders, the correlation linking ACA positivity to interstitial lung disease may be affected by residual bias. Third, given our retrospective design based on historical electronic medical records, missing values were observed in several clinical indicators. Each analysis was performed based only on patients with complete data for the corresponding variables, and no data imputation was conducted. This approach may introduce potential selection bias and reduce the generalizability of our findings. Future multicenter prospective studies with larger sample sizes, full collection of relevant clinical covariates, and standardized data recording systems are needed to confirm our conclusions.

## Conclusion

This retrospective case–control study identified a unique clinical and immunological phenotype in ACA-positive RA patients, who are at a significantly higher risk of xerostomia, xerophthalmia, Raynaud’s phenomenon, ILD, and cholestatic liver injury, with ACA positivity confirmed as an independent risk factor for these extra-articular manifestations. These findings highlight the clinical importance of ACA-based phenotypic stratification in RA, recommending routine screening for pulmonary, hepatic, and exocrine gland dysfunction in ACA-positive RA patients to facilitate early intervention. As a valuable serological marker, ACA may aid in the precise differential diagnosis of ACA-positive RA from other RA subtypes and connective tissue disease overlap syndromes. This study is limited by its single-center retrospective design, small sample size, lack of objective exocrine gland function assessments, and long-term follow-up data. Future multicenter prospective studies are needed to validate these findings and clarify the prognostic value of ACA positivity in RA. Further mechanistic studies are also required to explore the cellular and molecular pathways underlying ACA-mediated organ injury, which may support the development of personalized management strategies for ACA-positive RA patients.

## Data Availability

The original contributions presented in this study are included in this article/supplementary material, further inquiries can be directed to the corresponding authors.
